# Suspected *Legionella* Transmission from a Single Donor to Two Lung Transplant Recipients — Pennsylvania, May 2022

**DOI:** 10.15585/mmwr.mm7237a1

**Published:** 2023-09-15

**Authors:** Shannon McGinnis, Rebecca J. Free, Jacqueline Burnell, Sridhar V. Basavaraju, Trevor Kanaskie, Elizabeth J. Hannapel, Nottasorn Plipat, Kimberly Warren, Chris Edens

**Affiliations:** ^1^Bureau of Epidemiology, Pennsylvania Department of Health; ^2^Division of Healthcare Quality Promotion, National Center for Emerging and Zoonotic Infectious Diseases, CDC; ^3^Division of Infectious Diseases, Temple University Hospital, Philadelphia, Pennsylvania; ^4^Philadelphia Department of Public Health, Philadelphia, Pennsylvania; ^5^Division of Bacterial Diseases, National Center for Immunization and Respiratory Diseases, CDC.

**Summary**

**What is already known about this topic?**

Legionnaires disease is a severe pneumonia caused by *Legionella* bacteria. Comorbidities, including recent organ transplantation, increase the risk for infection.

**What is added by this report?**

In June 2022, two cases of Legionnaires disease were reported in patients, each of whom had received a lung transplant from the same donor, who had drowned in a river. Epidemiologic, environmental, and laboratory evidence suggest that the source of infection was likely the transplanted lungs.

**What are the implications for public health practice?**

Clinicians caring for patients who received organs from donors who drowned in fresh water should consider infection with *Legionella* in patients who develop postoperative complications. Prompt diagnosis and treatment of Legionnaires disease increases the likelihood of a full recovery.

## Abstract

In July 2022, the Pennsylvania Department of Health received two reports of laboratory-confirmed Legionnaires disease in patients who had recently received lung transplants from the same donor at a single Pennsylvania hospital. The donor’s cause of death was freshwater drowning in a river, raising suspicion of potential donor-derived transmission, because *Legionella* bacteria naturally live in fresh water. Further investigation of patients receiving other organs from the same donor did not identify additional legionellosis cases. Health care–associated infection caused by water exposure at the hospital was also evaluated as a potential source of infection and was found to be unlikely. Hospital water quality parameter measurements collected during May–June 2022 were within expected ranges and no water disruptions were noted, although no testing for *Legionella* was performed during this period. Notifiable disease data did not identify any other Legionnaires disease cases with exposure to this hospital within the 6 months before or after the two cases. Although laboratory testing did not confirm the source of recipient infections, available data suggest that the most likely source was the donor lungs. This cluster highlights the need for increased clinical awareness of possible infection with *Legionella* in recipients of lungs from donors who drowned in fresh water before organ recovery.

## Investigation and Results

In July 2022, the Pennsylvania Department of Health (PADOH) received reports of two cases of laboratory-confirmed Legionnaires disease in patients with exposure to the same Philadelphia hospital. Further investigation confirmed that each of the two patients had undergone transplantation of a single lung from the same donor before disease onset. Because of the possibility of a transplant-associated infection with *Legionella*, the hospital notified the Organ Procurement and Transplantation Network (OPTN) and initiated an investigation by OPTN’s ad hoc Disease Transmission Advisory Committee (DTAC). CDC, as a member of DTAC, led the investigation to determine whether the infections were transmitted through transplanted organs and to identify other patients who were potentially at risk.

The donor was a man aged 30–39 years who fell into a river and was submerged for ≥5 minutes. Despite resuscitation efforts, he sustained anoxic brain injury, which led to determination of brain death. Organ recovery occurred within 7 days of the drowning event. At the time, exposure to *Legionella* was not suspected, and no testing for *Legionella* was performed on any donor specimens before or after organ recovery.

The first Legionnaires disease case was identified in a woman aged 70–79 years (patient A) who received a right lung transplant in May 2022. Nine days after transplantation, the patient’s laboratory results revealed an elevated white blood cell count and acute anemia, which prompted imaging studies. A computed tomography (CT) scan was performed based on these blood test results. The CT scan identified dense consolidation in the middle lobe of the donor lung, which evolved into a cavitary lesion during the subsequent week (Figure). A bronchoalveolar lavage specimen collected during early June tested positive for *Legionella* species other than *Legionella pneumophila* by nucleic acid amplification. Doxycycline treatment was initiated immediately after *Legionella* was identified in the lavage specimen, and the patient fully recovered. No further testing was performed to identify the species or rule out the presence of multiple *Legionella* species.

**FIGURE:**
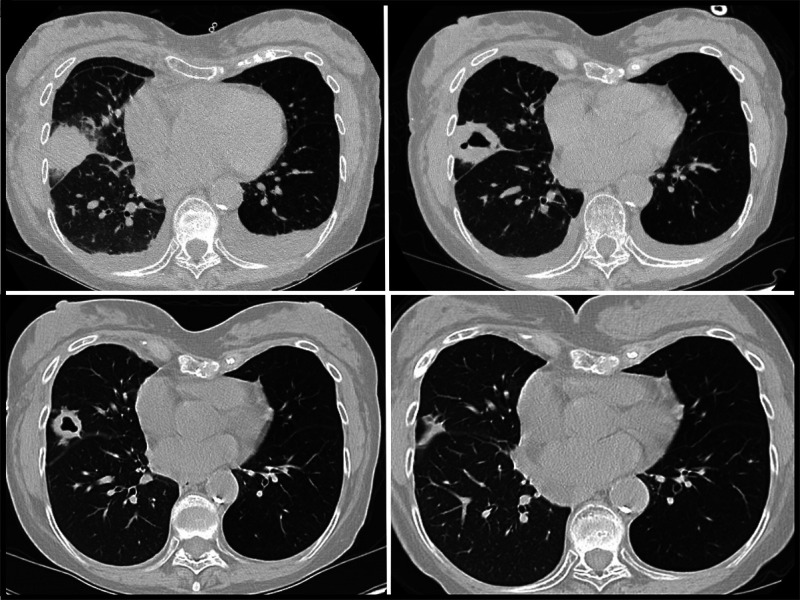
Computed tomography studies of the chest of patient A, who experienced infection with *Legionella* sp. (other than *Legionella* *pneumophila*) after receipt of a transplanted right lung from a donor who had drowned in fresh water, showing dense consolidative opacity with surrounding ground glass opacification (postoperative day 9) (A), thick-walled cavitation (postoperative day 16) (B), resolving lesion with thinning of cavity walls (postoperative day 29) (C), and continuing improvement (postoperative day 119) (D) — Philadelphia, Pennsylvania, May 2022

The second case occurred in a man aged 60–69 years (patient B) who received a left lung transplant on the same day and from the same donor as did patient A. Patient B experienced multiple postoperative complications, including the need for extracorporeal membrane oxygenation and renal replacement therapy, and he received antibiotic treatment with doxycycline starting on postoperative day 15. Ground glass–appearing opacities (a nonspecific sign that might represent infection) were noted in the donor lung after a CT scan performed 24 days after transplantation. After the first case was disclosed, a sputum specimen was collected during early June and tested positive for *L. pneumophila* by culture at a commercial laboratory. Although the patient experienced an initial clinical recovery, after a prolonged hospital stay, he died approximately 6 months after the transplant surgery due to respiratory failure secondary to a mucous plug.

Three additional recipients received transplanted organs from the same donor, including heart, liver, and right kidney. The heart, liver, and lung transplants occurred on the same day; the kidney transplant occurred 1 day later. The heart recipient had numerous nonspecific complications (none suggestive of legionellosis) after the transplant; no testing for *Legionella* was performed. The liver recipient had few postoperative complications, and a urinary antigen test result for *Legionella* was negative. The kidney recipient had few complications, but no evidence of postoperative infection; no testing for *Legionella* was performed. This activity was reviewed by CDC and was conducted consistent with applicable federal law and CDC policy.*

## Public Health Response

After notification of the two cases of Legionnaires disease, the Philadelphia Department of Public Health (PDPH) and PADOH requested water quality parameter and testing records for *Legionella* from the transplant hospital. Records collected during May–June 2022 were reviewed, including water management program data from both the potable water system and multiple cooling towers operated by the hospital. The cooling tower records indicated operation consistent with a well-functioning water management program.^†^ Potable water records collected during this period indicated that disinfectant was detected at all tested points of use. Routine testing of water samples for *Legionella* was not part of the facility’s water management program.

CDC attempted to identify any remaining clinical specimens from the donor or the organ recipients for testing for *Legionella*; however, no relevant specimens were available. Specimens relevant to testing for *Legionella* are not required to be collected or saved from organ donors. CDC and PADOH attempted to retrieve clinical isolates or any remaining clinical specimens from either lung transplant recipient and were similarly unsuccessful. The specimens collected from the patients either did not result in isolates after testing or were discarded by the commercial testing laboratory before additional testing could be performed.

PDPH and PADOH conducted retrospective and prospective active case finding by reviewing reportable disease surveillance data to ascertain whether any other persons with cases of Legionnaires disease were exposed to the transplant hospital during this period. No cases were identified during the 6 months before or after the transplant events.

## Discussion

*Legionella* bacteria are found naturally in freshwater environments, can survive under a wide range of environmental conditions, and typically grow best in warm water at temperatures of 77°F–113°F (25°C–45°C) (*1*). Exposure to water outside of human-made systems is not generally considered a risk factor for Legionnaires disease, which typically occurs after inhalation of water droplets containing *Legionella* (although aspiration is a recognized route of infection) (*2*). Before the cases described in this report were observed, infections with *Legionella* attributed to aspiration resulting from near-drowning incidents have been documented (*3*).

Legionnaires disease incidence has increased substantially during the past decade, reaching a peak of 2.71 cases per 100,000 persons in 2018 (*4*). Most cases are not associated with a known source, although approximately 18% have a reported health care facility–associated exposure (*5*). The Centers for Medicare & Medicaid Services requires that all acute care hospitals design and implement a water management program to reduce the growth and spread of *Legionella*.^§^ An effective water management program, along with strict adherence to infection control guidance, remain the best means to prevent health care–associated Legionnaires disease (*6*). Although solid organ transplantation is known to increase the risk for infection with *Legionella*, likely due to required immunosuppressive therapy, transmission via the organs themselves has not previously been reported (*2*). As with all infections in transplant recipients, prompt identification is critical to limit morbidity and mortality.

Despite a lack of confirmatory clinical or genomic evidence, three factors suggest that the transplanted lungs were the likely source of infection in the two cases presented in this report. First, different *Legionella* species were identified in the two patients. This can potentially be explained by infections derived from the donor’s exposure to river water, which might contain a larger diversity of *Legionella* species compared with potable water (*7*). Second, a review of records from the transplant facility indicated that water parameters were within expected ranges and the hospital did not report any disturbances to the building’s water system, recent changes in water quality parameters, or other events that might have increased the risk for infection with *Legionella* during this period. Finally, no other cases of legionellosis were reported from this facility within the 12-month period surrounding the two reported cases and no other possible sources of exposure were identified. Given the tight clustering in time, identification of additional cases would be expected if the source was the hospital facility’s water system or cooling towers.

### Limitations

The findings in this report are subject to at least three limitations. First, clinical specimens were not available from the donor for testing for *Legionella*. As a result, the presence of infection with *Legionella* in the donor before organ donation could not be confirmed. Second, specimens from the patients were not available for additional laboratory analyses which might have better characterized the species and serotypes present. In addition, the heart and kidney transplant recipients did not receive any *Legionella*-specific testing. Finally, the transplant hospital’s building water system and cooling towers were not tested for *Legionella* before or immediately after the surgical procedures. Although the hospital’s water parameter data indicate a well-maintained system, presence of *Legionella* bacteria in the water system at the time of the transplants cannot be ruled out.

### Implications for Public Health Practice

This report adds to the understanding of microbial risk assessment among recipients of organs from donors who died from drowning. Previous studies have documented bacterial and fungal pathogen transmission to transplant recipients when the donors have drowned, which have sometimes resulted in outcomes including bacterial and fatal fungal infections (*8*–*10*). The present findings suggest that clinicians caring for patients who receive organs from donors who experienced freshwater drowning also should maintain a higher index of suspicion for legionellosis, even in organ recipients without classic clinical symptoms. In such patients, posttransplant antimicrobials could be tailored to include agents that combat atypical waterborne organisms. When an unexpected donor-derived infection is suspected, providers are required to report the case to the United Network for Organ Sharing or OPTN for investigation by DTAC and to public health authorities for expedited evaluation and identification of potentially infected organs and tissues.^¶^ Prompt assessment by astute clinicians can result in more rapid diagnosis and treatment of Legionnaires disease, which requires organism-specific testing, thereby increasing the likelihood of a full recovery.

## Footnotes

* 45 C.F.R. part 46.102(l)(2), 21 C.F.R. part 56; 42 U.S.C. Sect. 241(d); 5 U.S.C. Sect. 552a; 44 U.S.C. Sect. 3501 et seq.

† https://www.cdc.gov/legionella/wmp/control-toolkit/index.html^§^ https://www.cms.gov/Medicare/Provider-Enrollment-and-Certification/SurveyCertificationGenInfo/Policy-and-Memos-to-States-and-Regions-Items/Survey-And-Cert-Letter-17-30-

¶
https://optn.transplant.hrsa.gov/media/eavh5bf3/optn_policies.pdf
